# Association between alcohol consumption during pregnancy and risks of congenital heart defects in offspring: meta-analysis of epidemiological observational studies

**DOI:** 10.1186/s13052-016-0222-2

**Published:** 2016-02-03

**Authors:** Zhongyuan Wen, Di Yu, Weiyan Zhang, Changfeng Fan, Liang Hu, Yu Feng, Lei Yang, Zeyu Wu, Runsen Chen, Ke-jie Yin, Xuming Mo

**Affiliations:** Department of Cardiothoracic Surgery, Nanjing Children’s Hospital, Nanjing Medical University, Nanjing, 210008 China; Department of Neurology, Center for the Study of Nervous System Injury, Washington University School of Medicine, St. Louis, MO 63110 USA

**Keywords:** Congenital heart defects, Alcohol drinking, Pregnancy, Meta-analysis

## Abstract

**Background:**

To explore the association between maternal alcohol consumption and/or binge drinking and congenital heart defects (CHDs), we conducted a meta-analysis for more sufficient evidence on this issue.

**Methods:**

We searched Medline, EMBASE, and the Cochrane Library from their inceptions to December 2014 for case-control and cohort studies that assessed the association between maternal alcohol consumption and CHD risk. Study-specific relative risk estimates were calculated using random-effect or fixed-effect models.

**Results:**

A total of 19 case-control studies and 4 cohort studies were included in the meta-analysis. We observed a null association between maternal alcohol consumption during pregnancy and the risk of CHDs. Even in the analysis of different trimesters of pregnancy, we found little association between the two.

**Conclusions:**

This meta-analysis suggests that maternal alcohol consumption is modestly not associated with the risk of CHDs. However, further investigation is needed to confirm this conclusion.

## Background

Congenital heart defects (CHDs) are the most common group of congenital malformations, affecting nearly 1 % of live births throughout the world [1]. CHDs account for approximately one-third of all congenital anomalies and are the leading noninfectious cause of death in the first year of life [2]. Although massive breakthroughs have been achieved in cardiovascular diagnostics and cardiothoracic surgery over the past century, leading to the increased survival of newborns with CHDs, the etiology of most congenital heart defects is still unknown.

A number of chromosomal anomalies, certain maternal illnesses, and prenatal exposures to specific therapeutic drugs are recognized risk factors. It is difficult to establish the role of a single factor because in many cases, the cause of a defect is believed to be multifactorial, including environmental teratogens with genetic and chromosomal conditions [3]. Maternal alcohol consumption is associated with a variety of harmful effects to the fetus, as demonstrated by the range of impairments present in fetal alcohol syndrome [4]. Various clinical signs have been described, which led to the classification of different degrees of embryopathy, ranging from patients with minor symptoms, the so-called “alcohol effects”, to the most severely affected individuals [5]. Up to one-third of affected individuals have congenital cardiac disease [6].

A study by Jones *et al*. [7] was one of the first to report the association between maternal alcohol consumption and CHDs. However, the evidence since then has been mixed, with some studies showing positive associations and others providing null results. CHDs include distinct subtypes (e.g., conotruncal defects, left ventricular outflow track defects, septal defects), and there is potential for etiologic heterogeneity. Binge drinking is defined as consuming ≥5 alcoholic drinks at one sitting [8].

Because the matter of whether there is an association between maternal alcohol consumption and/or binge drinking and congenital heart defects remains uncertain, we conducted this meta-analysis for more sufficient evidence on this issue.

## Methods

A computerized literature search was conducted in MEDLINE, EMBASE, and the Cochrane Library from their inceptions to December 1, 2014, by two independent investigators (Wen and Yu). We searched relevant studies using the following medical subject heading terms and/or text words: “congenital heart defect”, “heart abnormalities”, “CHD”, and “heart malformation” in combination with “alcohol”, “drinking”, “maternal alcohol consumption”, “maternal drinking”, “periconceptional drinking”, and “binge drinking”. In addition, we performed a broader search on environmental teratogens and CHDs and checked the reference lists of retrieved articles and relevant review articles to identify additional relevant studies.

### Eligibility criteria

We selected articles that (1) were original epidemiologic studies (i.e., case-control, cohort), (2) were published in the English language, (3) examined the association between maternal alcohol consumption any time during pregnancy and CHDs overall or any one of the CHD subtypes in infants, (4) reported relative risk or odds ratios (RRs or ORs) and associated 95 % confidence intervals (CIs) or had raw data available, (5) defined CHDs or one of the CHD subtypes as an outcome, and (6) provided exposure information. If the articles were duplicated or were from the same study population, the article with a larger sample size was included. Non-peer-reviewed articles, ecologic assessments, correlation studies, experimental animal studies and mechanistic studies were excluded.

### Data extraction

One study author (Wen) first screened studies by title and abstract and made exclusions based on the eligibility criteria. The studies that met the inclusion criteria were independently reviewed by two authors (Wen and Yu) to retrieve the information of interest including study characteristics (i.e., authors, year of publication, geographic region, periods of data collection, study design, case classification, control definition, sample size, source of exposure data, drinking status, levels of drinking, exposure period during pregnancy, and adjusted/matched variables) and to record reported effect estimates and associated 95 % CIs as well as raw data if effect estimates were not available. Discrepancies between the authors were resolved by discussion.

The corresponding risk estimates (including RRs and ORs) and 95 % CIs were extracted from each study for CHDs overall, CHD subtypes and the period of maternal alcohol consumption during pregnancy. We selected the main confounder-adjusted RRs or ORs whenever possible. Otherwise, unadjusted effect estimates were extracted from each study. We conducted meta-analyses for specific CHD subtypes (i.e., sub-analyses) if at least two studies had available data. The RRs were used as the common measure of association across studies. ORs were transformed into RRs according to the formula RR = OR/[(1-P_0_) + (P_0_ × OR)] where P_0_ stands for the incidence of CHDs [9]. Because of the low incidence of CHDs, ORs could be considered as RRs.

### Statistical analysis

Statistical analysis was based on comparing the highest alcohol consumption and binge drinking with the lowest consumption (which could have included women who did not drink). We calculated summary RR estimates and 95 % CIs using both fixed- and random-effects models for CHDs overall and for the CHD subtypes.

We first tested for heterogeneity across studies using Cochran *Q* and *I*^*2*^ statistics [10]. If there was evidence of heterogeneity (*P* < 0.05 or *I* ^*2*^≧ 50 %), we used a random-effects model, which provided a more appropriate summary effect estimate between heterogeneous study-specific estimates. If the Cochran *Q* and *I*^*2*^ statistics showed no evidence of heterogeneity, we used a fixed-effects analysis, applying inverse variance weighting to calculate summary RR estimates [11].

Meta-analyses of total maternal alcohol consumption and binge drinking were both included. Subgroup analysis of CHDs was conducted by CHD subtype (e.g., conotruncal defects, left ventricular outflow track defects, septal defects), study design (cohort and case-control studies), geographic region (Europe and America) and study adjustments (smoking, maternal age and ethnicity). In the main analyses that examined the association between maternal alcohol consumption and the risk of CHDs overall, we calculated a summary effect estimate for CHDs overall limited to the studies that examined the association between maternal periconceptional drinking (i.e., 3 months before pregnancy through the first trimester) and CHDs.

Publication bias was evaluated by generating funnel plots for a visual examination, conducting correlation and regression tests for significance, and using Egger’s linear regression [12] and Begg’s rank correlation [13] methods. A *P* value of <0.05 for the two aforementioned tests was considered representative of significant statistical publication bias. All statistical analyses were performed using STATA (version 11.0; StataCorp, College Station, Texas, USA).

## Results

### Literature search

**Fig. 1:**
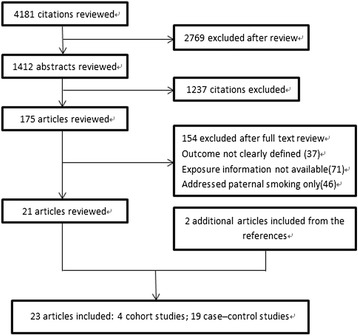
Study selection procedures. Study selection procedures for a meta-analysis of alcohol consumption during pregnancy and the risk of congenital heart defects (CHDs) in offspring

The search strategy generated 4181 citations, of which 23 were identified in the final analysis (Fig. 1). All of the studies were published from 1989 to 2014. There were 19 case-control studies [14–32] and 4 cohort studies [33–36].

### Study characteristics

The main characteristics of the included studies are shown in Table 1. As is shown, 13 studies [15–20, 24, 25, 27, 28, 30–32] were conducted in the United States, 7 in Europe [14, 21–23, 26, 29, 34], and 3 in other regions (Canada and Australia) [33, 35, 36]. A wide range of exposure periods was examined, with 10 studies reporting the mother’s drinking or binge drinking during the first trimester of pregnancy, including 1 to 3 months before conception [14, 17–19, 24, 25, 27, 28, 31, 33]; however, 13 studies did not specify the months of exposure during pregnancy. Additionally, 5 studies provided estimates on the association between maternal alcohol consumption and CHDs adjusted for a range of covariates (e.g., smoking, coffee consumption, maternal age, ethnicity, education, occupation) [17, 19, 33, 34, 36], and 18 studies reported only unadjusted estimates (Table 1). The studies derived their cases from various birth defect registries [18–23, 29, 35], and the control subjects were randomly selected from birth certificates or hospital records or matched to cases by birth region or birth month [16, 18]. For some studies, cases were derived from a sample of live-born infants, whereas for other studies, cases were also identified from stillbirths, neonatal deaths, and elective terminations.

**Table 1:** Summary of the studies included in the meta-analysis

First author, year	Region	Period	Study design	No. of cases/controls^a^	period	OR/RR	95 % CI	Adjustment variables^b^	Outcome
Bean, 2011	USA	2001–2004	CC	566/552	1^st^ month gestation	1.07	0.58–1.98	No	AVSD
						1.03	0.48–2.23		ASD
						0.49	0.19–1.22		VSD
					2^nd^/ 3^rd^ month gestation	0.64	0.19–2.21		AVSD
						1.50	0.40–5.67		ASD
						1.08	0.27–4.36		VSD
Botto, 2000	USA	1968–1980	CC	958/3029	During pregnancy	1.00	0.86–1.15	No	CHDs
Carmichael, 2003	USA	1987–1988	CC	207/481	1 month before to 3^rd^ month gestation	1.35	0.84–2.19	B, D, F, K, I	TGA
						1.24	0.77–1.98		TOF
Cedergren, 2002	Sweden	1982–1996	CC	269/524	Early pregnancy	0.95	0.69–1.31	No	CHDs
Ewing, 1997	USA	1981–1989	CC	641/3549	During pregnancy	0.95	0.78–1.15	No	VSD
Fixler, 1998	USA		CC	89/82	3 months before to 3^rd^ month gestation	1.23	0.67–2.28	No	CHDs
Grewal, 2008	USA	1999–2003	CC	323/700	During pregnancy	1.21	0.85–1.73	No	Conotruncal defects
Hobbs, 2010	USA	1998–2006	CC	572/363	1 month before to 3^rd^ month gestation	0.84	0.63–1.12	No	CHDs
Kuciene, 2009	Lithuania	1999–2005	CC	187/643	During pregnancy	1.39	0.87–2.25	No	CHDs
Larsen, 2011	Denmark	1996–2002	Cohort	477/ 86783	During pregnancy	1.24	0.97–1.59	A, E, F, H, P	VSD
						0.89	0.66–1.21		ASD
Liu, 2013	Canada	2002–2010	Cohort	4123/22365	During pregnancy	1.88	1.74–2.04	A, B, E, F, H, L, O	CHDs
McDonald, 1992	Canada	1982–1984	Cohort	318/87389	First trimester	0.96	0.78–1.19	A, B, D, F, G	CHD
Malik, 2008	USA	1997–2002	CC	3067/3947	1 month before to 3^rd^ month gestation	0.93	0.85–1.03	O	CHDs
Martinez-Frias, 2004	Spain		CC	4705/4329	During pregnancy	0.96	0.78–1.19	No	CHD
Mateja, 2012	USA	1996–2005	CC	237/948	3 months before pregnancy	1.01	0.61–1.70	No	CHDs
O’Leary, 2013	Australia.	1983–2007	Cohort	277/61370	During pregnancy	1.40	0.91–1.98	No	VSD
						2.86	1.22–4.93		ASD
						2.01	0.58–4.07		Conotruncal defects
Smedts, 2009	Netherlands	2008	CC	276/324	During pregnancy	1.05	0.75–1.47	No	CHDs
Steinberger, 2002	USA	1981–1989	CC	237/3572	During pregnancy	1.00	0.10–7.70	No	Single Ventricle
Tikkanen, 1991	Finland	1982–1984	CC	573/1055	First trimester	1.29	1.05–1.59	No	CHDs
Tofs, 1999	USA	1991–1993	CC	80/605	First trimester	0.90	0.50–1.40	No	CHDs
						1.30	0.70–2.40		AVSD
						1.00	0.20–2.40		TOF
						0.80	0.50–1.40		ASD
						0.60	0.30–1.20		VSD
Van Beynum, 2010	Netherlands	1996–2005	CC	611/2401	During pregnancy	1.05	0.85–1.29	No	CHDs
Williams, 2004	USA	1968–1980	CC	122/3029	3 months before to 3^rd^ month gestation	1.02	0.71–1.47	No	VSD
Yauck, 2004	USA	1997–1999	CC	245/2780	During pregnancy	2.10	1.10–4.20	No	CHDs

*CC* case-control study, *CHDs* congenital heart defects, *VSD* Ventriculap Septal Defect, *ASD* Atrial Septal Defect, *TOF* Tetralogy of Fallot, *TGA* D-Transposition of the Great Arteries, *HLHS* Hypoplastic Left Heart Syndrome, *COA* Coarctation of the Aorta, *AVSD* Atrioventricular Septal Defect

^a^Reported number of cases and control subjects with available exposure information

^b^Adjustment variables: A maternal age, B maternal race/ethnicity, C marital status, D maternal education, E parity, F smoking, G coffee consumption, H infant’s year/month of birth, I intake of multivitamin, J stress, K folic acid intake/dietary folate, L infant gender, M maternal body mass index, N family history of congenital heart defects, O maternal residence, P maternal occupation, Q insurance,

### Maternal alcohol consumption and CHDs

**Fig. 2:**
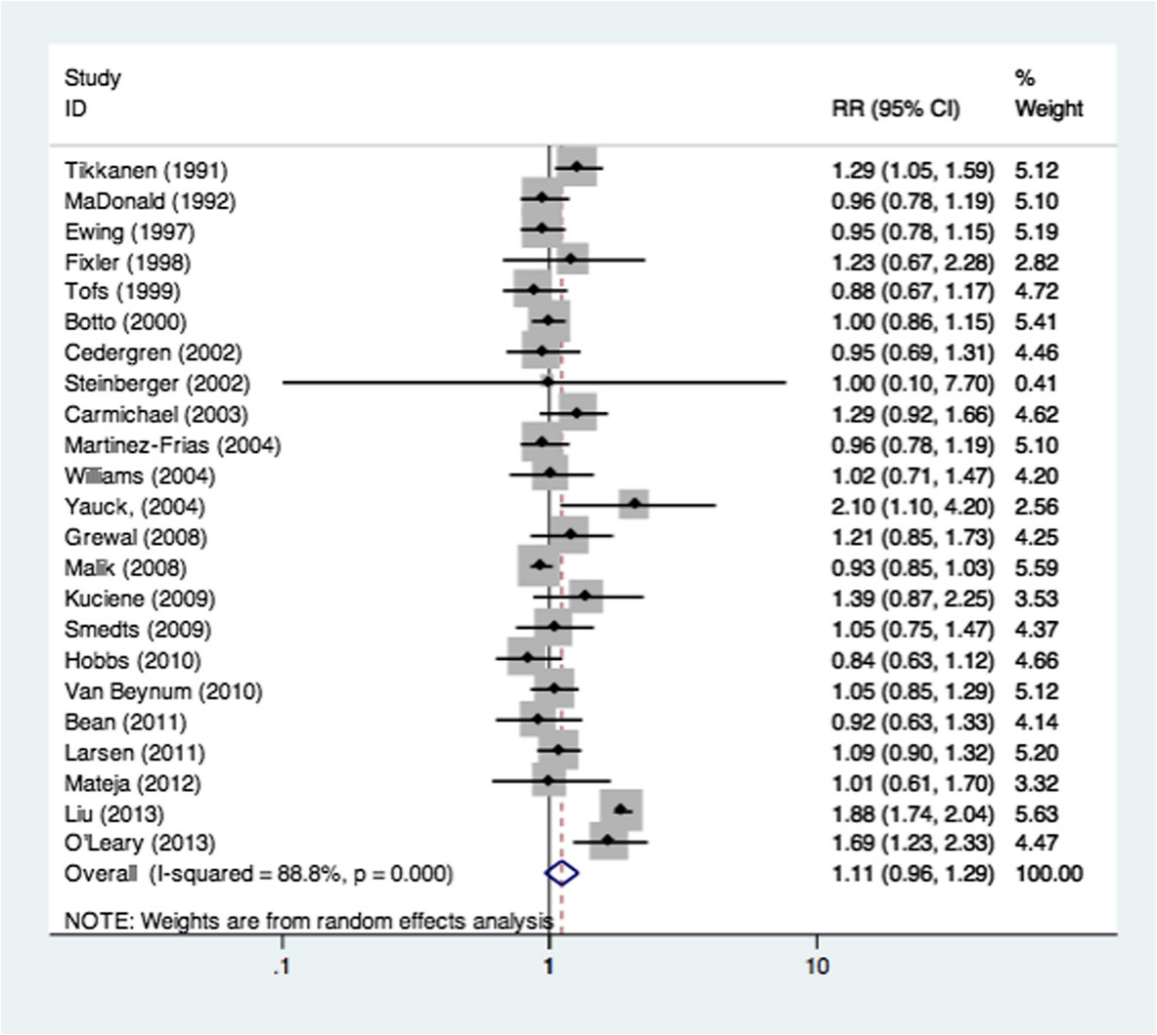
Pooled Relative risk (RR). RR estimates for the association between maternal alcohol consumption and the risk of CHDs. Meta-analysis random effects estimates were used. The sizes of the squares reflect the weighting of the included studies. Bars represent 95 % confidence intervals (CIs). The center of the diamond indicates the summary effect; the left and right points of the diamond indicate the 95 % confidence interval

**Fig. 3:**
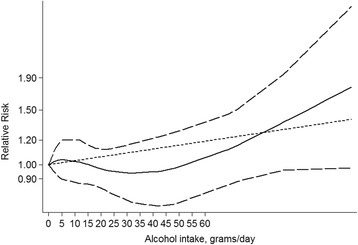
The dose-response of relative risk and alcohol intake: The dose-response of relative risk and alcohol intake: the corresponding risk of CHDs did not increase with the increase of alcohol consumption. The full line indicates the relative risk of CHDs along with the alcohol intake (grams/day); the dashed lines indicate the 95 % confidence interval

Overall, 23 studies evaluated the association between maternal alcohol consumption during pregnancy and CHDs as a group in a total of 19,160 CHD cases (Table 1). We found that maternal alcohol consumption may have no association with an increased risk of CHDs (RR =1.11, 95 % CI = 0.96–1.29) (Fig. 2). The results were consistent with the overall summary measure when the analyses were restricted to case-control studies (RR = 1.00, 95 % CI = 0.95-1.06) or cohort studies (RR = 1.35, 95 % CI = 0.93-1.97). When we turned to the period of maternal alcohol consumption during pregnancy, the statistics showed that maternal drinking during different times of pregnancy had no impact on the incidence of CHDs. Specifically, 6 studies focused on women who drank during pre-pregnancy and confirmed that no association was found between maternal drinking and CHD risk (RR = 0.95, 95 % CI = 0.88–1.04) (Table 2). Another 22 studies also looked at women who drank during pregnancy, but no results showed that maternal drinking during pregnancy was associated with CHDs (RR = 1.12, 95 % CI = 0.96–1.30) (Table 2). Additionally, Egger’s test (*P* = 0.156) showed no evidence of publication bias for the period of maternal alcohol consumption during pregnancy. In the subgroup analyses by geographic region, we observed null results among both European (RR = 1.09, 95 % CI = 0.99–1.19) and North American (RR = 1.10, 95 % CI = 0.89–1.36) populations. In the adjustment models, we chose smoking as important, but unfortunately, a null result was found for smoking (RR = 1.284, 95 % CI = 0.817–2.016). Eight other studies were about the dose-response data on maternal alcohol consumption and risk of CHDs. It showed that with the increase of alcohol consumption, the corresponding risk of CHDs did not increase (Fig. 3). Most of these results showed that the amount of maternal drinking was not associated with CHDs except in 2 studies: Martinez-Frias’s research reported that women who drank more than 116 grams a day possibly had a 1093 % increased risk of CHDs (RR = 11.93, 95 % CI = 1.62–246.00), and Williams’ report showed that maternal alcohol consumption of more than 22 grams per day was associated with a 213 % increased risk of CHDs (RR = 3.13, 95 % CI =1.19–8.22) (Table 3).

**Table 2:** Summary risk estimates of the association between maternal alcohol comsuption and CHD risk in offspring

Subgroup analysis	No. of studies	Summary RR (95 % CIs)	P*	I^2^ (%)	P**
Summary pooled estimate	23	1.11(0.96–1.29)	<0.001	88.8	
Design					0.025
Case-control	19	1.00(0.95–1.06)	0.260	16.0	
Cohort	4	1.35(0.93–1.97)	<0.001	94.4	
Geographical region					0.447
North America	15	1.10(0.89–1.36)	<0.001	92.1	
Europe	7	1.09(0.99–1.19)	0.449	0.0	
Australia	1	1.69(1.23–2.33)	-	-	
Period of pregnancy					0.343
Pre-pregnancy or until pregnancy is known	6	0.95(0.88–1.04)	0.426	0.0	
During pregnancy	22	1.12(0.96–1.30)	<0.001	89.3	
Trimester 1	22	1.12(0.96–1.30)	<0.001	89.3	
Trimester 2	12	1.22(0.98–1.52)	<0.001	90.9	
Trimester 3	12	1.22(0.98–1.52)	<0.001	90.9	
Adjusted for smoking					0.108
Yes	4	1.27(0.86–1.87)	<0.001	94.6	
No	19	1.04(0.96–1.13)	0.042	39.0	

*Abbreviations*: *RR* relative risk, *CI* confidence interval

*p-value for heterogeneity within each subgroup

**p-value for heterogeneity between subgroups with meta-regression analysis

**Table 3:** Characteristics of 8 observational studies with dose-response data on maternal alcohol consumption and risk of CHDs

First author, year	Region	Period	Study design	No. of cases/controls^a^	period	Average dose (grams/d)^b^	OR/RR	95 % CI	Adjustment variables^c^	Outcome
Carmichael, 2003	USA	1987–1988	CC	207/481	1 month before to 3^rd^ month gestation	0	1.00	1.00–1.00	B, D, F, K, I	Conotruncal defects
						0.9	1.20	0.80–1.70		
						3	1.60	0.80–3.00		
Ewing, 1997	USA	1981–1989	CC	641/3549	During pregnancy	0	1.00	1.00–1.00	No	VSD
						18	0.92	0.73–1.15		
						48	0.91	0.71–1.78		
						90	1.25	0.85–1.83		
Fixler, 1998	USA		CC	89/82	3 months before to 3^rd^ month gestation	0	1.00	1.00–1.00	No	CHD
						18	1.36	0.57–3.25		
						42	1.20	0.48–3.02		
						69	1.11	0.42–2.96		
Larsen, 2011	Denmark	1996–2002	Cohort	477/ 86783	During pregnancy	0	1.00	1.00–1.00	A, E, F, H, P	VSD
						1.7	1.22	0.90–1.66		
						3.4	1.38	0.83–2.28		
						5.1	1.11	0.54–2.23		
						0	1.00	1.00–1.00		ASD
						1.7	1.03	0.73–1.47		
						3.4	0.45	0.18–1.11		
						5.1	0.66	0.27–1.62		
Martinez-Frias, 2004	Spain		CC	4705/4329	During pregnancy	0	1.00	1.00–1.00	No	CHD
						15	1.01	0.76–1.34		
						24	1.66	0.35–8.73		
						32	0.82	0.59–1.15		
						72	1.99	0.32–15.60		
						116	11.93	1.62–246.00		
McDonald, 1992	Canada	1982–1984	Cohort	318/87389	First trimester	0	1.00	1.00–1.00	A, B, D, F, G	CHD
						2.9	0.91	0.70–1.20		
						8.7	0.96	0.60–1.50		
						17.9	1.24	0.70–2.20		
Williams, 2004	USA	1968–1980	CC	122/3029	3 months before to 3^rd^ month gestation	0	1.00	1.00–1.00	No	VSD
						4.3	0.95	0.65–1.39		
						12	1.22	0.43–3.45		
						22	3.13	1.19–8.22		

*CC* case-control study, *CHDs* congenital heart defects, *VSD* Ventriculap Septal Defect, *ASD* Atrial Septal Defect, *TOF* Tetralogy of Fallot, *TGA* D-Transposition of the Great Arteries, *HLHS* Hypoplastic Left Heart Syndrome, *COA* Coarctation of the Aorta, *AVSD* Atrioventricular Septal Defect

^a^Reported number of cases and control subjects with available exposure information

^b^Converted into pure ethanol in grams from the original data

^c^Adjustment variables: A maternal age, B maternal race/ethnicity, C marital status, D maternal education, E parity, F smoking, G coffee consumption, H infant’s year/month of birth, I intake of multivitamin, J stress, K folic acid intake/dietary folate, L infant gender, M maternal body mass index, N family history of congenital heart defects, O maternal residence, P maternal occupation, Q insurance, R cases and controls matched on birth hospital/geographic region, birth month/age, race, or sex

## Discussion

**Fig. 4:**
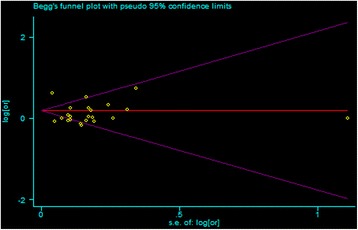
Begg’s test. Begg’s test of studies examining the association between maternal alcohol consumption and the risk of CHDs

To our knowledge, this is the first meta-analysis to report on the association between maternal alcohol consumption and CHDs. Although the studies included in our analysis varied in terms of case definition, control selection, and exposure assessment, the associations were largely consistent in the sub-analyses. Our findings indicated that maternal alcohol consumption during pregnancy might have no association with the risk of CHDs. Additionally, statistically significant heterogeneity was detected (*Q* = 46.52, *P* < 0.001, *I*^*2*^ = 88.8 %), although no publication bias was indicated from Egger’s test (*P* = 0.156) while Begg’s test (*P* = 0.037) indicated a publication bias (Fig. 4). Because of the existence of heterogeneity, the power of Begg’s test lowed. Therefore we conducted the subgroup analyses and meta-regression analyses (Table 2). In the sub-analyses of design types, geographical region, period of pregnancy and adjusted for smoking, the results were almost consistent with the pooled RR. Meta-regression analysis showed that the different study design might result in the heterogeneity. Moreover, when findings were stratified by geographic reign, null results were found among American and European populations, although a rate of 69 % was found among Australian populations. However, the study on Australian populations was conducted in only one paper by one author [31], and there were 277 cases from 1983–2007 in his report. He observed positive associations between maternal alcohol consumption during pregnancy and the CHD subtypes he analyzed: Ventricular septal defect (VSD) (RR = 1.36, 95 % CI = 1.14–1.63) and Atrial septal defect (ASD) (RR = 1.77, 95 % CI = 1.27–2.46); conotruncal defects (RR = 1.50, 95 % CI = 0.98–2.30). O’Leary attempted to demonstrate that maternal drinking was associated with different types of CHDs, but the number of ASD cases he found was 60, only 0.3 % of the exposed group, and the rate of conotruncal defects was 0.1 % as well. A smaller sample size could be why this result differed from others. However, there is another possibility that is worth our attention. The differences between the American, European and Australian natures of alcohol consumption could also have caused these effects. As we know, different wine has different components. For example, beer is made from wheat and *Humulus lupulus*, whisky is mainly made from *Hordeum vulgare*, brandy is made from fruits such as grapes, and so on. Criqul MH found the so-called “French Paradox”, that the French have a relatively low incidence of coronary heart disease while having a diet that is relatively rich in saturated fats [37]. He indicated that wine made from grapes could be the primary explanation for the phenomenon. However, we found very few statistics regarding the ingredients of different wine, and from our point of view, this question deserves to be studied in the future. Among those statistics that found that maternal alcohol consumption had a positive association with CHD risk, Martinez-Frias reported that consumption of more than 116 grams per day could increase CHD risk [29]. We must here highlight that this study had only 67 cases and 20 controls, which is quite small compared with the other sample sizes, and this authors report also had no adjustment variables; this dose of alcohol consumption could lead to a number of diseases that could have an impact on CHD risk. The Williams study had the same problem as well. Based on the adjustment results, the potentially important confounding factor of smoking was excluded in the analysis of alcohol consumption. However, there was no association between maternal alcohol consumption and CHDs following adjustment for smoking, indicating that smoking has little influence on CHDs. However, Lee *et al*. [38] confirmed the association between maternal smoking and increased risk of CHDs by meta-analysis. The influence of maternal smoking on CHD risk needs further study.

The result of our meta-analysis is in reality rather confusing. As we all know, alcohol consumption has a number of effects on our health. However, our research shows that maternal alcohol consumption during pregnancy may have no association with the risk of CHDs. Ethyl alcohol as the main component of wine has been suggested to play a positive role in heart disease. We speculate that a small amount of alcohol may have little influence in increasing the risk of CHDs. However, these statistics do not intend to say that maternal drinking is safe; the fact is that even low levels of prenatal alcohol exposure, such as in a single dose, can produce the birth defect termed fetal alcohol syndrome (FAS), and as many as 54 % of live-born children with FAS present with some form of cardiac anomalies [39], e.g., septal defects and pulmonary stenosis [20], that can lead to developmental challenges, ongoing medical care and death. Additionally, findings have shown that alcohol consumption during pregnancy may affect the Wnt/β-catenin signaling that allows for normal gene activation and cardiogenesis [40].

Our study has a number of strengths, including the large sample (n = 19,160) used to estimate the effect of maternal alcohol consumption on CHDs as a group. Additionally, because the suspected heterogeneous etiologies can affect CHD risk during different times in pregnancy, we divided pregnancy into stages; still, no association was found in any of the stages. However, our study must also be considered in light of certain limitations. First, our analysis is limited to studies published in English. Evidence of publication bias was found, and there was heterogeneity in the component studies, which could have been attributable to study design, study populations, analytic strategies or other unknown factors. Second, we derived most of our data from case-control studies, which may be more prone to information bias than cohort studies. Third, the consumption quantity in each study varied, including times/week, drinks/week, drinks/occasion, etc. The highest and lowest intakes varied across studies, and the highest intake in one study could have been similar to the median or lowest intake in another, which could have biased the overall results. Additionally, based on the different methods that were used to assess and report alcohol consumption across studies, we established the dose-response relationship between alcohol consumption and CHDs. It showed that the risk of CHDs did not influence with the increase of alcohol consumption, even binge drinking. Fourth, because of our inability to fully adjust for various confounders, associations between alcohol consumption and CHDs could be attributed to other factors, such as maternal smoking, ethnicity, age, and education. Fifth, our analysis included a wide range of exposure periods, but heart anomalies develop during weeks 2–7 of gestation [41]. We suspect that our including studies that assessed exposure beyond the critical period may have biased our result toward the null. That is, the summary results may be a misestimate of the relative risk of CHDs associated with alcohol consumption.

## Conclusions

In conclusion, our analysis indicates that maternal alcohol consumption has no association with CHD risk. However, the findings from our study need to be confirmed in future research in well-designed cohort or intervention studies. In addition, the underlying mechanisms call for further elucidation.

Although its effects are modest, drinking is relatively common among women of reproductive age and could have important public health consequences. Young women continue to drink although the adverse effects of drinking on reproductive health are known, and some women continue to drink even after they learn that they are pregnant. Decreased maternal alcohol consumption during pregnancy would result in improved reproductive outcomes and could contribute to reduced infant mortality and morbidity.

## Abbreviations

ASD: Atrial septal defect
CHDs: Congenital heart defects
CIs: Confidence intervals
FAS: Fatal alcohol syndrome
ORs: Odds ratios
RRs: Relative risks or risk ratios
VSD: Ventricular septal defect
